# Impact of endocrine therapy regimens for early-stage ER+/HER2- breast cancer on contralateral breast cancer risk

**DOI:** 10.1038/s41523-025-00746-7

**Published:** 2025-03-26

**Authors:** Swarnavo Sarkar, Clyde Schechter, Allison W. Kurian, Jennifer L. Caswell-Jin, Jinani Jayasekera, Jeanne S. Mandelblatt

**Affiliations:** 1 Department of Oncology, Georgetown University Medical Center and Cancer Prevention and Control Program, Lombardi Comprehensive Cancer Center, Washington, DC USA; 2 Department of Family and Social Medicine, Albert Einstein College of Medicine, New York City, NY USA; 3 Department of Medicine (Oncology) and Department of Epidemiology and Population Health, Stanford University School of Medicine, Stanford, CA USA; 4 Department of Medicine (Oncology), Stanford University School of Medicine, Stanford, CA USA; 5 Health Equity and Decision Sciences Laboratory, National Institute on Minority Health and Health Disparities, National Institutes of Health, Rockville, MD USA; 6 Georgetown Lombardi Institute for Cancer and Aging REsearch (I-CARE), Lombardi Comprehensive Cancer Center, Washington, DC USA

**Keywords:** Breast cancer, Epidemiology, Epidemiology

## Abstract

Endocrine therapy for breast cancer may reduce the risk of contralateral breast cancer (CBC). However, there are no published estimates quantifying the lifetime outcomes by age at primary diagnosis, regimen, or duration. Here, we adapted an established Cancer Intervention and Surveillance Network (CISNET) model to simulate life histories of multiple US female birth-cohorts diagnosed with stage 0-III ER+/HER2- breast cancer receiving different durations (none, 2.5, 5, 10 years) of two endocrine therapy regimens (aromatase inhibitors or tamoxifen; including ovarian-function suppression for premenopausal women). As expected, greater duration of endocrine therapy led to more avoided CBC cases, as did aromatase inhibitors over tamoxifen, but the numbers varied greatly by the age of diagnosis. The maximum number of CBC were avoided using 10-year aromatase inhibitor regimens (6.0 vs. 11.2 for no adjuvant therapy, per 100 women with ER+/HER2- breast cancer). For the 5-year aromatase inhibitors therapy, women <45 years had the largest reduction in CBC cases (5.0/100), which dropped to 2.7/100 for women at 75+ years. Quantification of the lifetime risk of CBC for specific endocrine therapy types and duration is helpful for weighing therapeutic options. The risk of breast cancer death has a larger weight, but inclusion of the risk of CBC increases the separation between different therapy options.

## Introduction

There has been a steady decline in US contralateral breast cancer incidence rates^1^, but these cancers remain the most prevalent second primary cancer among breast cancer survivors^2^. Endocrine therapy regimens for the primary breast cancer can both improve survival and reduce the risk of developing contralateral breast cancer among women with estrogen receptor positive (ER+) and human epidermal growth factor receptor negative (HER2-) breast primary cancers^3–7^, which constitute nearly two-thirds of all primary breast cancers^8^.

Clinical trials and large observational studies suggest that aromatase inhibitors may be more effective in reducing the rates of cancer-specific death, and contralateral breast cancer incidence compared to tamoxifen regimens^3,9^. Studies also suggest that women are more likely to discontinue hormonal regimens due to various adverse effects associated with these treatment^6,10–14^. Shorter durations of therapy may yield lower benefits from endocrine therapy, including reducing the risk of contralateral breast cancer^15–18^. Moreover, past studies have shorter follow-up inadequate sample size for sub-group analyses, and limited generalizability to women seen in real-world practice settings. Consequently, there are insufficient data on the possible variation of breast cancer outcomes over a woman’s lifetime considering her age at diagnosis, primary regimens and treatment durations to support shared clinical decision-making.

Simulation modeling from the Cancer Intervention and Surveillance Network (CISNET) has been extensively used to provide similar clinical decision-making aids for breast cancer (https://cisnet.cancer.gov/breast/). Recent contributions include a decision analysis for the US Preventive Task Force for screening protocols using mammography^19^, population-level effects of omitting chemotherapy guided by gene expression assay in node-positive breast cancer^20^, and screening strategies for women with pathogenic variants^21^. The CISNET methodology enables adoption of a wide spectrum of clinical trial and meta-analysis data to determine the efficacy of breast cancer screening and treatment for different subpopulations, which is needed to estimate the cumulative impact of age at diagnosis, primary therapy regimen and duration on contralateral breast cancers.

In this study, we adapted an established CISNET breast cancer simulation model, developed at Georgetown University and Albert Einstein College of Medicine (Model G-E), to synthesize high-quality nationally representative data and simulate lifetime risks of contralateral breast cancer, primary breast cancer, and other-cause survival in U.S. women diagnosed with ER+/HER2- breast cancer by age group, endocrine therapy regimen and duration^22,23^. The results are intended to inform clinical guidelines and support shared decision-making about the use of endocrine therapy to prevent contralateral breast cancer.

## Results

**Fig. 1:**
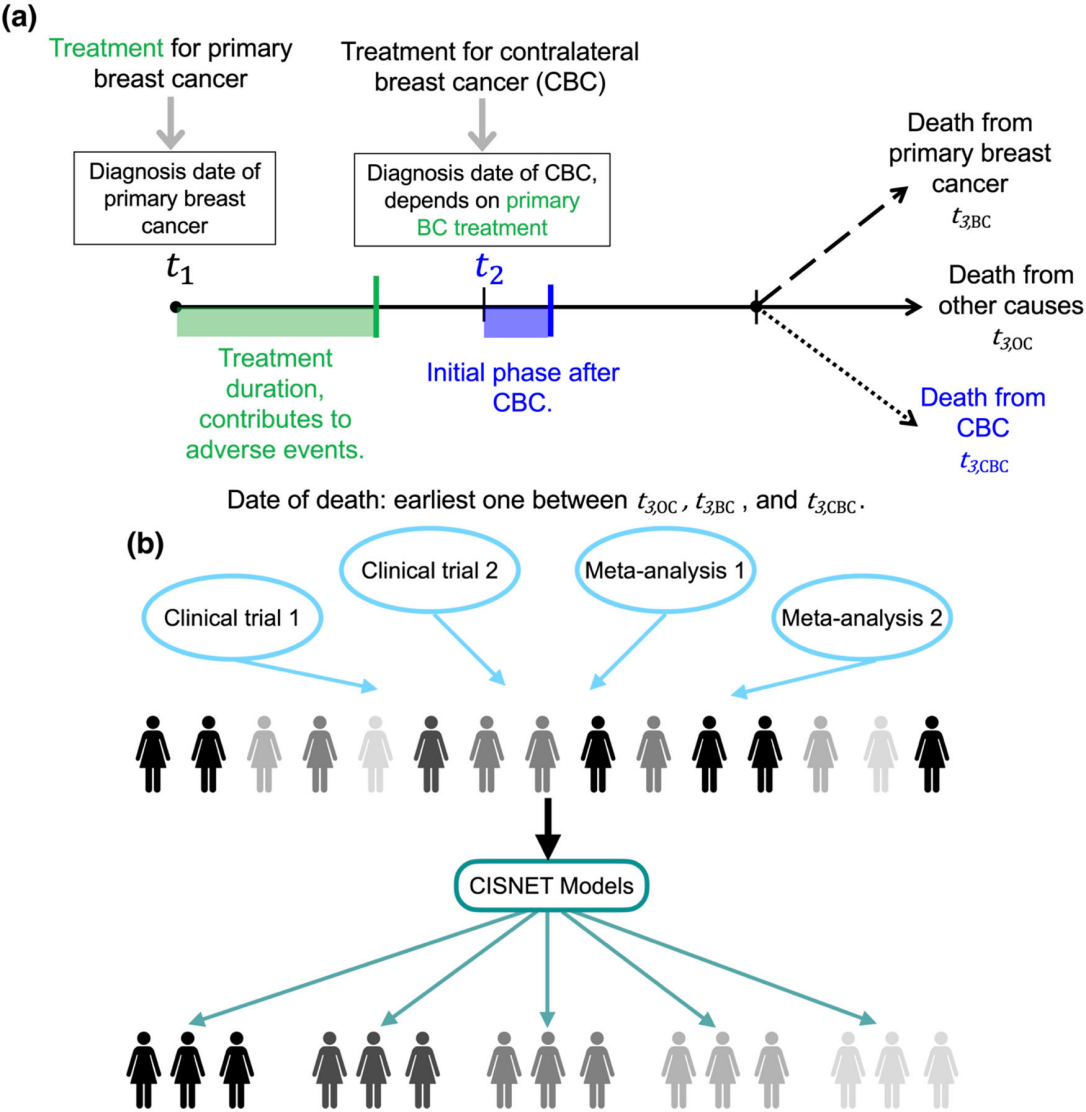
Schematic representation of simulating contralateral breast cancer and refinement of clinical decision-making using CISNET models. **a** For this work we updated Model GE to estimate the time interval to a contralateral breast cancer event (*t*_*2*_) from the date of diagnosis of the primary breast cancer (*t*_*1*_). This time interval between the primary and the contralateral breast cancer (contralateral breast cancer) is estimated depending on the type and duration of endocrine therapy used to treat the primary breast cancer and the year and the age at diagnosis of the primary breast cancer. Once a contralateral breast cancer event occurs, we apply treatment at *t*_*2*_ and estimate remaining survival time. **b** Role of CISNET models for refining clinical decision-making aids for subpopulations of women. Clinical trials and meta-analyses data cover heterogeneous population who can vary by age, subtype, and other clinically relevant factors, which is represented using the glyphs of different shades in the image. CISNET computational models adapt the cumulative data from clinical trials and meta-analyses to quantify clinical estimates for narrowly categorized subpopulations of women.

**Fig. 2:**
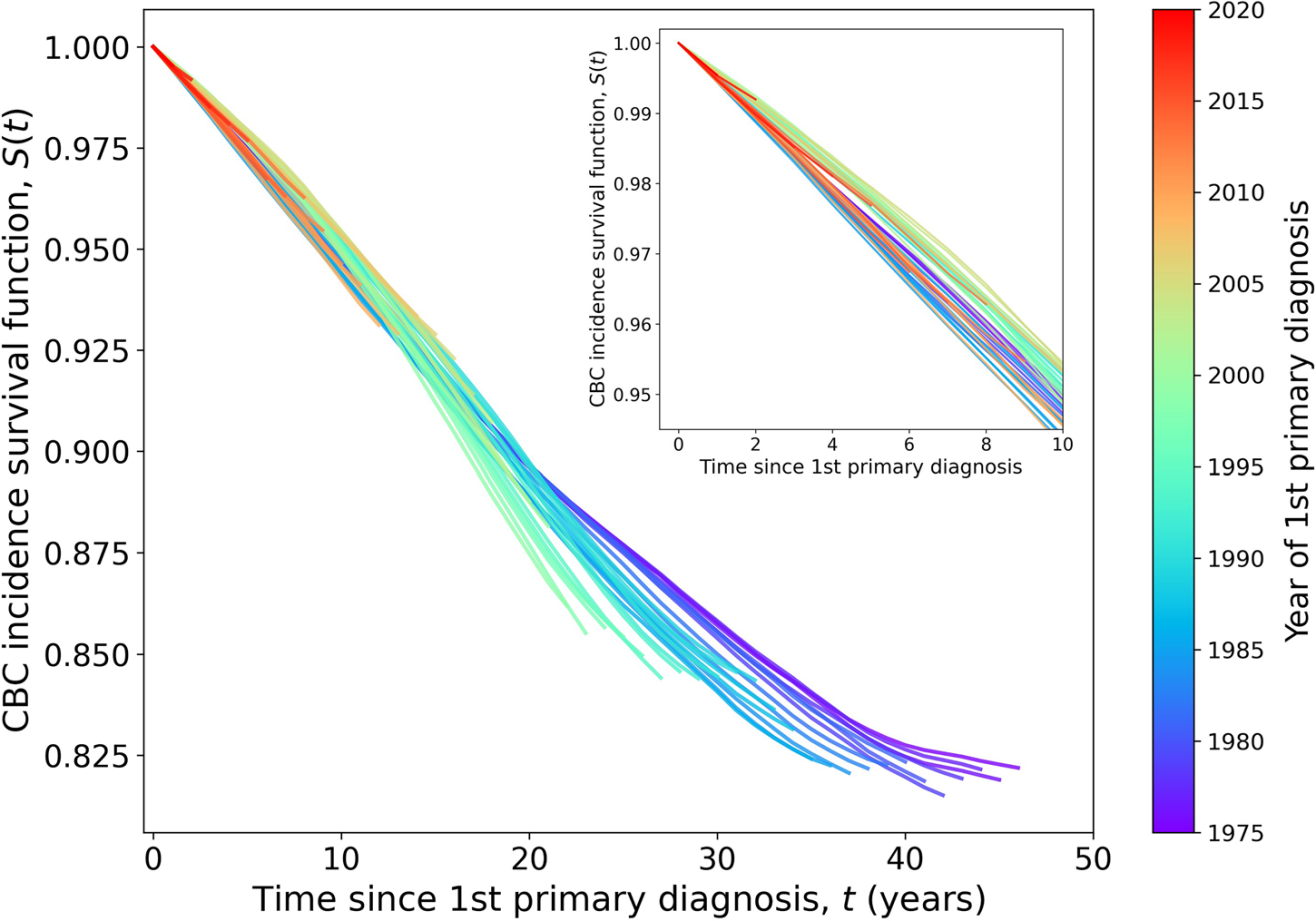
Cumulative risk of contralateral breast cancer in the US female breast cancer survivors. Kaplan-Meier survival functions for contralateral breast cancer incidence using the contralateral breast cancer incidence rate data from SEER (Supplementary Fig. 2). The survival functions are blue-shifted for earlier years of first primary diagnosis, i.e., the bluest curve is for contralateral breast cancer incidence among 1st primary breast cancer patients diagnosed in 1975. The reddest curve is for the year 2020. Earlier years of 1st primary diagnosis have more years elapsed for the occurrence of contralateral breast cancer, and therefore those curves are longer. The inset figure zooms in on contralateral breast cancer incidence survival functions for the first 10 years since the diagnosis of the 1st primary.

For this analysis, we modified Model G-E to include the incidence and treatment of future contralateral breast cancers, conditional on age of diagnosis, treatment regimen, and duration, for the primary breast cancer (Fig. 1a, Table 1, and “Methods”). We focused on metachronous contralateral breast cancers, defined as second primary breast cancer with the opposite laterality and occurring at least 6 months after the first primary breast cancer. Building on the CISNET methodology, this modified Model G-E uses clinical trial and meta-analysis data that cumulatively cover a heterogeneous population of women, varying by age groups, endocrine therapy type, and duration, and computationally estimates the rate of contralateral breast cancer and breast cancer mortality for narrowly-defined subpopulations (Fig. 1b). The primary new input is a set of contralateral breast cancer incidence functions that quantify the probability of a contralateral breast cancer starting from the date of the first primary breast cancer diagnosis (Fig. 2 and “Methods”).

**Table 1:** Model input parameters

Input parameter	Data		Data sources
Contralateral breast cancer incidence functions	*S*(years after 1st primary diagnosis; age at 1st BC, year of diagnosis)		SEER for the contralateral breast cancer incidence functions^60^.
Contralateral breast	Chemotherapy	0.7	Netherlands Cancer Registry and Pathology
cancer incidence	Endocrine therapy	0.46	database (PALGA) for the HRs^9^.
HRs^a^	Chemotherapy + Endocrine therapy	0.35	
For specific ET and duration			
Overall survival HRs	Aromatase inhibitors + OFS, 2.5 years	0.88	5-year HRs from SOFT-TEXT trials^3,6^,
Age < 50 years	Aromatase inhibitors + OFS, 5 years	0.47	and Breast International Group 1-98
(premenopausal)	Aromatase Inhibitors + OFS, 10 years	-	data^32,54^.
	Aromatase Inhibitors, 2.5 years	0.71	2.5-year HRs from non-adherence HRs from
Age ≥ 50 years	Aromatase Inhibitors, 5 years	0.54	the Kaiser Permanente of Northern
(postmenopausal)	Aromatase Inhibitors, 10 years	0.53	California data analysis^56^.
Contralateral breast cancer incidence HRs	Aromatase Inhibitors + OFS, 2.5 years	0.63	10-year HRs from MA.17R trial^15^.
Age < 50 years	Aromatase Inhibitors + OFS, 5 years	0.49	HRs that were reported with tamoxifen
(premenopausal)	Aromatase Inhibitors + OFS, 10 years	-	therapy as the referent group were
	Aromatase Inhibitors, 2.5 years	0.68	converted to the no adjuvant therapy
Age ≥ 50 years	Aromatase Inhibitors, 5 years	0.37	referent group using the tamoxifen therapy
(postmenopausal)	Aromatase Inhibitors, 10 years	0.2	HRs in the EBCTCG data^18,24^.

^a^Model-GE uses treatment-specific hazard ratios for overall survival, which has been reported in previous CISNET Breast Working Group publications^19,53^. The contralateral breast cancer incidence rate for a specific adjuvant therapy is a function of both the hazard ratio for overall survival and the hazard ratio for the contralateral breast cancer. Data for tamoxifen and tamoxifen + ovarian function suppression regimens are in Supplementary Tables 1 and 2.

### Validation

**Fig. 3:**
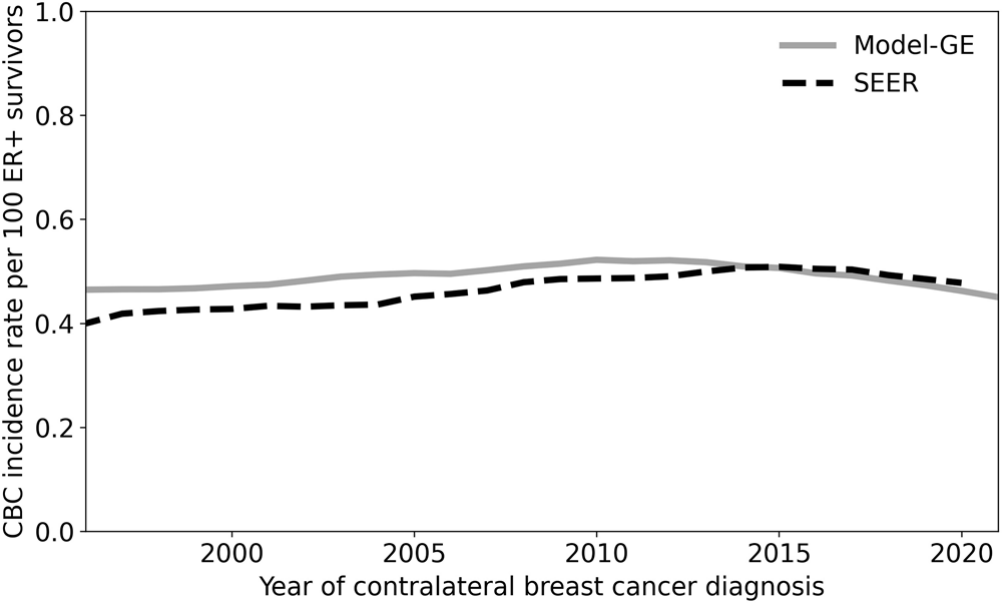
Comparison of contralateral breast cancer incidence rates from Model G-E and SEER. The x-axis is the year of diagnosis of contralateral breast cancer and y-axis is the contralateral breast cancer incidence rate per 100 women with primary, non-metastatic ER+ breast cancer. The total incidence rate of contralateral breast cancers from all adjuvant therapy categories from Model G-E (gray line) and the SEER contralateral breast cancer incidence rate using the data from SEER registry 12 (1992–2020) (dashed black line). Only data for women with metachronous contralateral breast cancer occurring at least 6 months after the primary breast cancer were used to compute the incidence rates.

The model generated contralateral breast cancer incidence rate was similar to observed SEER rates for women with primary early-stage ER+ tumors, differing from SEER by less than 7% in any year, which is less than 0.029 cases per year per 100 women (Fig. 3). The model results were also compared to SEER for contralateral breast cancer incidence rates among women with primary ER+/HER2- tumors (Supplementary Fig. 4). SEER began reporting HER2- status of women with breast cancer only from 2010, and a 10-year interval is insufficient to estimate the contralateral breast cancer incidence rates.

### Regimen

The absolute number of lifetime contralateral breast cancers varied by the type and duration of endocrine therapy for the primary ER+/HER2- breast cancer (Table 2). For all regimens, the longer the duration of treatment, the fewer the number of contralateral breast cancers. Aromatase inhibitors (with ovarian function suppression for premenopausal survivors) (vs. no endocrine therapy) lead to greater reductions in the number of contralateral breast cancers than tamoxifen (vs. no endocrine therapy) across all treatment durations. For example, compared to no endocrine therapy, 10-year use of aromatase inhibitors (with ovarian function suppression for premenopausal survivors) reduced the number of contralateral breast cancers from 11.2 to 6.0 per 100 women with primary ER+/HER2 cancers (5.2 contralateral cancers avoided), while 10 years of tamoxifen for reduced the number to 6.5 (4.7 contralateral cancers avoided).

**Table 2:** Contralateral breast cancers per 100 ER+/HER2- breast cancer patients for different durations of tamoxifen, tamoxifen + ovarian function suppression (OFS), and aromatase inhibitor^a^ regimens, overall and in different age groups

	Absolute number of contralateral breast cancers expected per 100 women based on age and treatment duration			
Age range (years)	No adjuvant therapy	2.5 years	5 years	10 years^b^
Results per 100 women with 1st primary ER+/HER2- cancer treated with tamoxifen regimens				
All ages	11.2	9.8	7.9	6.5
<45	16.7	15.1	13.4	12.0
45–49	14.0	12.4	11.0	9.8
50–64	11.8	10.3	8.0	6.6
65–74	9.0	7.7	5.9	4.6
75+	6.6	5.8	4.4	3.3
Results per 100 women with 1st primary ER+/HER2- cancer treated with tamoxifen + OFS regimens				
<45	16.7	14.5	12.5	-
45–49	14.0	11.8	10.6	-
Results per 100 women with 1st primary ER+/HER2- cancer treated with aromatase inhibitor regimens^c,d^				
All ages	11.2	8.9	7.0	6.0
<45	16.7	12.8	11.7	-
45–49	14.0	11.0	10.0	-
50–64	11.8	9.5	7.1	5.7
65–74	9.0	7.2	5.3	4.2
75+	6.6	5.4	3.9	3.0

^a^Aromatase inhibitors therapy for pre-menopausal patients (age < 50 years) always include ovarian function suppression (OFS); OFS is optional for when treating premenopausal women with tamoxifen.

^b,c^Stage 0 or DCIS premenopausal first primary cases were treated with tamoxifen therapy for a maximum of 5 years, even in the simulation scenarios for 10-year durations of endocrine therapy.

^d^Aromatase inhibitors with OFS was applied to premenopausal breast cancer patients (<50 years) for 5 years in the simulation of the population for 10 years of AI therapy. Postmenopausal patients received the full 10 years of AI therapy.

### Age

Across all age groups, 10 years of endocrine therapy was associated with the greatest reduction in the number of contralateral breast cancers (Table 2). However, for women diagnosed at the age of 75+ years, the difference between 5- and 10 years of aromatase inhibitors therapy was only 0.9 contralateral breast cancer avoided per 100 women. In contrast, for women between 50 and 64 years, the difference between 5- and 10 years of aromatase inhibitor therapy was 1.4 additional contralateral breast cancers avoided per 100 women with primary ER+/HER2- cancer.

### Duration

**Fig. 4:**
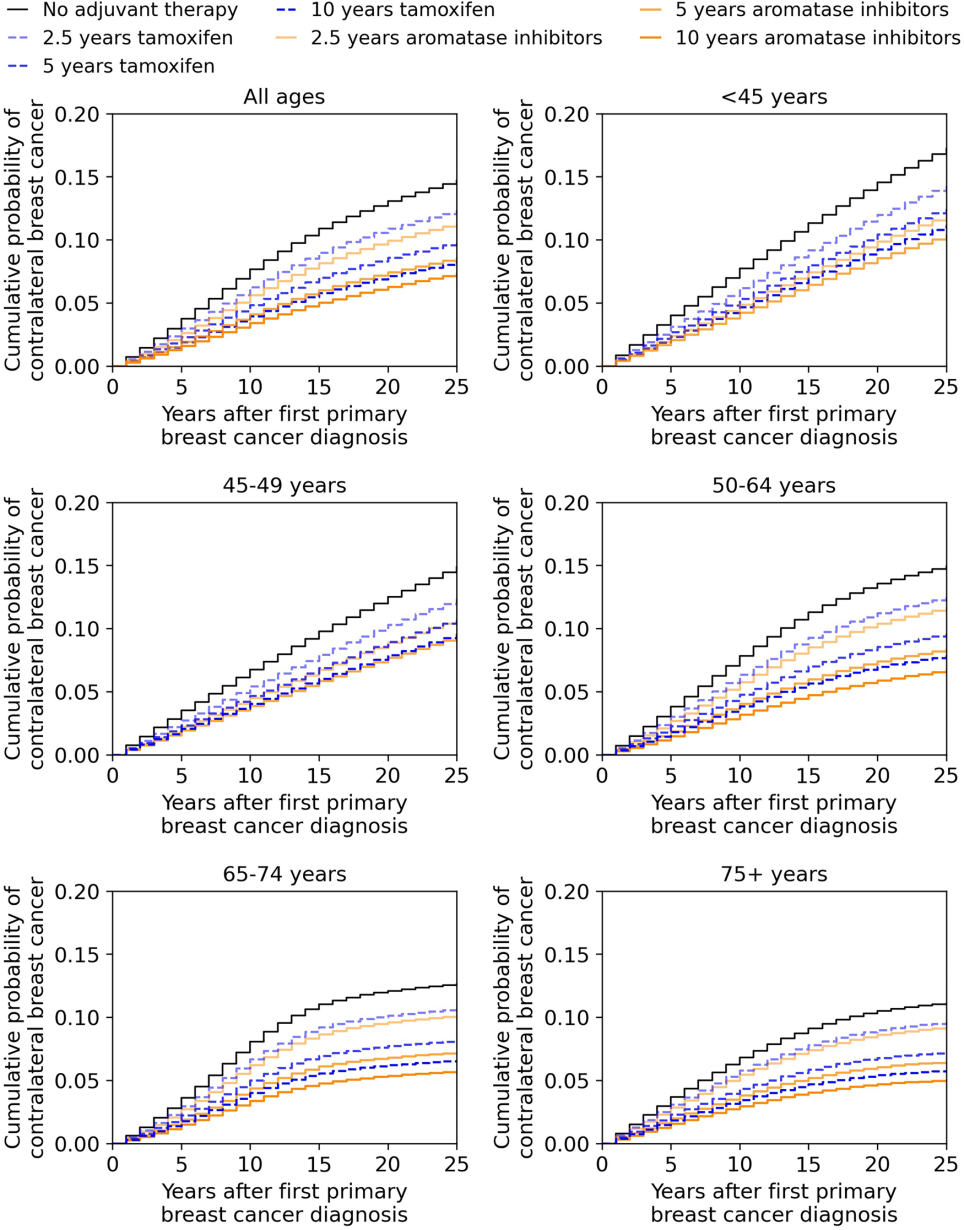
Cumulative risk of contralateral breast cancer depending on the age at first primary diagnosis. Each subplot shows the cumulative probability of contralateral breast cancer as a function of the years after the first primary diagnosis. The age-range at the title of the subplots specifies the age of a woman at the first primary diagnosis of ER+/HER2- breast cancer. The black line corresponds to the no adjuvant therapy scenario. The dashed blue lines are for tamoxifen regimens and the orange lines are for aromatase inhibitors regimens. Shorter durations of tamoxifen and aromatase inhibitors regimens are depicted using more translucent lines. Aromatase inhibitors with ovarian function suppression therapy was applied only for 5 years for the premenopausal breast cancer patients. The aromatase inhibitors for 10 years line in the subfigure for “All ages” uses only 5 years of endocrine therapy for the premenopausal women with breast cancer.

For postmenopausal women with breast cancer, the 25-year cumulative risk of contralateral breast cancer was reduced by 6–10% for the 10-year aromatase inhibitors therapy (vs. no adjuvant therapy) (Table 3 and Fig. 4). In premenopausal women, 5-year aromatase inhibitor therapy reduced the 25-year cumulative risk of contralateral breast cancer by an additional 1–2% compared to 5-year tamoxifen therapy. In postmenopausal women, incomplete aromatase inhibitor therapy (2.5 years) had almost twice the cumulative risk of contralateral breast cancer (vs. 10-year therapy). For older women (75+ years), the 5-year cumulative risk of contralateral breast cancer with 2.5-years of endocrine therapy was only 1% higher that 5-year therapy.

**Table 3:** Cumulative risk of contralateral breast cancer depending on the age at the diagnosis of the first ER+/HER2- breast cancer and the time interval after the primary breast cancer

		Cumulative risk (in %) of contralateral breast cancer after the primary ER+/HER2- breast cancer						
Age (years)	Time since primary cancer diagnosis (years)	No adjuvant therapy	For Tamoxifen therapy of different durations			For Aromatase Inhibitors^a^ therapy of different durations		
			2.5 years	5 years	10 years^b^	2.5 years	5 years	10 years^c,d^
	5	4.8	3.7	3.2	2.8	2.9	2.5	-
	10	8.5	6.8	5.9	5.1	5.4	4.7	-
<45	15	12.0	9.8	8.5	7.5	7.9	6.9	-
	20	15.2	12.5	10.9	9.7	10.3	9	-
	25	17.7	14.7	12.9	11.5	12.3	10.7	-
	5	4.2	3.3	2.8	2.4	2.7	2.3	-
	10	7.4	5.9	5.1	4.4	4.9	4.3	-
45–49	15	10.3	8.4	7.3	6.4	7.2	6.2	-
	20	13.0	10.7	9.3	8.2	9.3	8	-
	25	15.3	12.7	11.1	9.8	11.1	9.7	-
	5	4.6	3.7	2.7	2.2	3.4	2.4	1.9
	10	8.6	7	5.2	4.2	6.9	4.8	3.7
50–64	15	11.8	9.7	7.4	6	8.9	6.3	4.9
	20	13.9	11.5	8.8	7.2	9.7	6.9	5.4
	25	15.2	12.6	9.7	8	10.2	7.2	5.7
	5	4.5	3.7	2.8	2.2	3.2	2.2	1.7
	10	8.8	7.3	5.5	4.4	6.4	4.5	3.4
65–74	15	11.3	9.5	7.2	5.7	8.2	5.8	4.5
	20	12.2	10.3	7.8	6.3	9.0	6.4	5.0
	25	12.7	10.7	8.2	6.6	9.4	6.7	5.2
	5	4.3	3.7	2.7	2.2	3.4	2.4	1.9
	10	7.4	6.3	4.7	3.7	5.9	4.1	3.2
75+	15	9.5	8.1	6.1	4.9	7.7	5.4	4.2
	20	10.7	9.2	6.9	5.5	8.8	6.2	4.8
	25	11.1	9.6	7.2	5.8	9.2	6.5	5.0

Cumulative risk (in %) is reported for different types and durations of endocrine therapy.

^a^Aromatase inhibitor therapy for pre-menopausal patients (age < 50 years) always includes ovarian function suppression.

^b,c^Stage 0 or DCIS premenopausal first primary cases were treated with tamoxifen therapy for a maximum of 5 years, even in the simulation scenarios for 10-year durations of endocrine therapy.

^d^Aromatase inhibitors with OFS was applied to premenopausal breast cancer patients (<50 years) for 5 years in the simulation of the population for 10 years of AI therapy. Postmenopausal patients received the full 10 years of AI therapy.

### Breast cancer deaths

Across all ages, a 2.5-year duration of aromatase inhibitors therapy resulted in more breast cancer deaths than 5-years, but the reduction in breast cancer deaths due to extending endocrine therapy duration from 5 to 10 years was small, especially for aromatase inhibitors regimens (Table 4). As expected, the largest number of breast cancer deaths can be prevented by completing 5 vs. 2.5-years of primary therapy with among women <45 years. The number of other-cause deaths per 100 women with primary ER+/HER2- cancers was higher for women taking 5-year or 10-year endocrine therapy because they were less likely to die of the primary breast cancer. Our estimate of the quality-adjusted life years (QALYs) due to different duration of aromatase inhibitors therapy showed that 10-year regimen can provide a gain in QALYs (vs a 5-year regimen) only if there is low prevalence and low disutility from adverse events (Methods, Supplementary Table 3 and Supplementary Fig. 5).

**Table 4:** Lifetime breast cancer deaths per 100 ER+/HER2- breast cancer patients for different durations of tamoxifen and aromatase inhibitors^a^ regimens, overall and in different age groups

	Absolute number of breast cancer deaths expected per 100 women based on age and treatment duration				Absolute number of other cause deaths^b^ expected per 100 women based on age and treatment duration			
Age range (years)	No adjuvant therapy	2.5 years	5 years	10 years^c^	No adjuvant therapy	2.5 years	5 years	10 years^d^
Results per 100 women with 1st primary ER+/HER2- cancer treated with tamoxifen regimens								
All ages	21.9	16.9	15.1	14	45.9	48.6	49.6	51.2
<45	28.3	20.8	19.3	19.1	30.7	33.1	33.6	33.7
45–49	24.2	17.9	16.6	16.3	36.9	39.6	40.2	40.5
50–64	23.0	17.4	15.5	14.2	43.1	46.2	47.3	48.5
65–74	19.4	15.5	13.7	12.3	53.1	55.7	56.9	59.1
75+	16.2	13.7	12.2	10.6	59.3	61.2	62.3	65.1
Results per 100 women with 1st primary ER+/HER2- cancer treated with aromatase inhibitor regimens^e^								
All ages	21.9	17.7	13.4	13.2	45.9	48.0	50.6	50.7
<45	28.3	20.6	16.0	-	30.7	33.1	34.8	-
45–49	24.2	17.7	13.6	-	36.9	39.7	41.6	-
50–64	23.0	18.7	14.0	13.8	43.1	45.4	48.2	48.3
65–74	19.4	16.5	12.4	12.2	53.1	55.0	57.9	58.0
75+	16.2	14.6	11.0	10.8	59.3	60.4	63.2	63.3

^a^Aromatase inhibitor therapy for pre-menopausal patients (age < 50 years) always includes ovarian function suppression.

^b^Breast cancer deaths and other cause deaths for each treatment type and duration does not add up to 100 because results were tallied until the year 2050 and some women will not have died by that year.

^c,d^Stage 0 or DCIS premenopausal first primary cases were treated with tamoxifen therapy for a maximum of 5 years, even in the simulation scenarios for 10-year durations of endocrine therapy.

^e^Aromatase inhibitors with OFS was applied to premenopausal breast cancer patients (<50 years) for 5 years in the simulation of the population for 10 years of AI therapy. Postmenopausal patients received the full 10 years of AI therapy.

### Cumulative risks of contralateral breast cancers and deaths

**Fig. 5:**
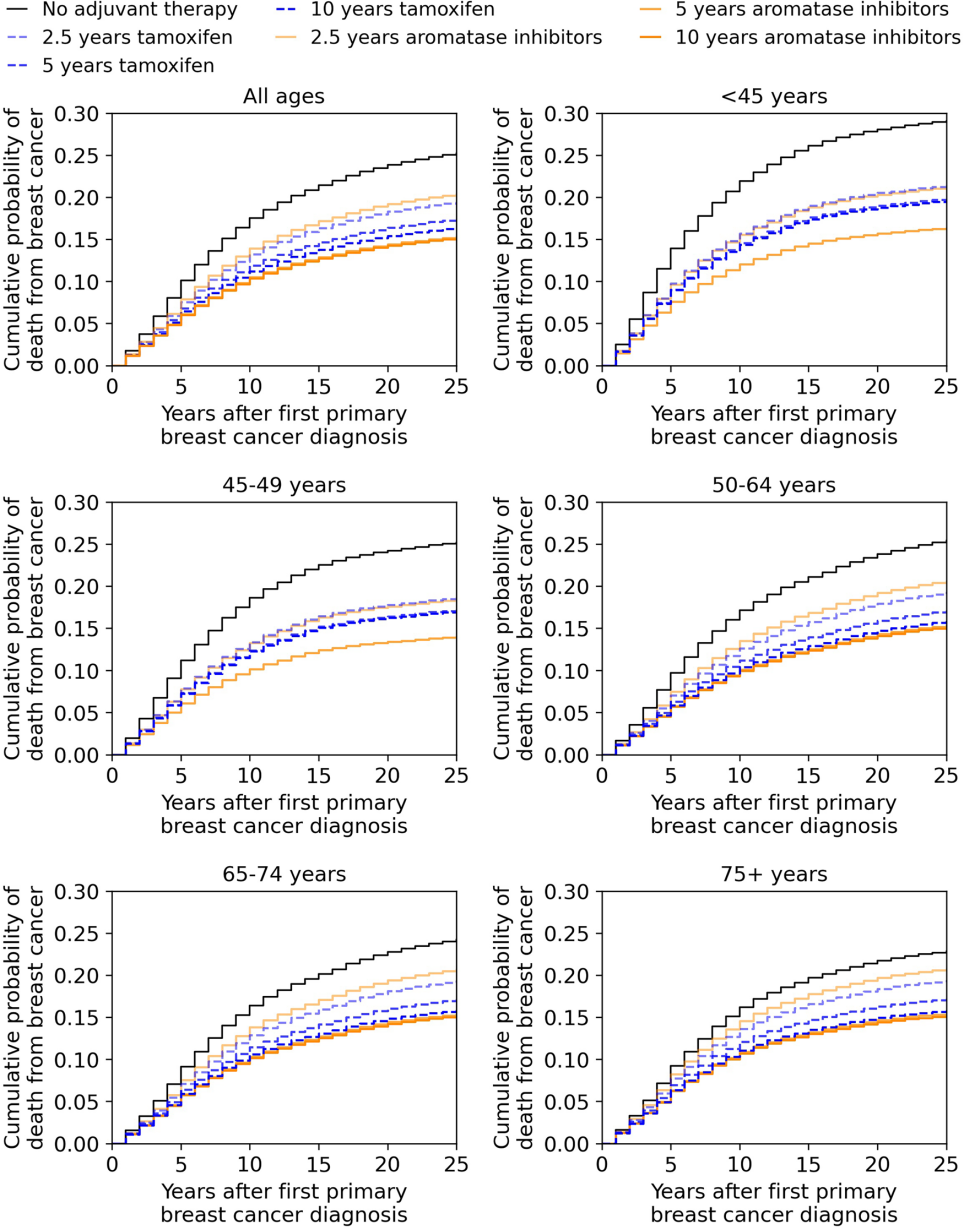
Cumulative risk of death from breast cancer depending on the age at first primary diagnosis. Each subplot shows the cumulative probability of death from breast cancer as a function of the years after the first primary diagnosis. The age-range at the title of the subplots specifies the age of a woman at the first primary diagnosis of ER+/HER2- breast cancer. The black line corresponds to the no adjuvant therapy scenario. The dashed blue lines are for tamoxifen regimens and the orange lines are for aromatase inhibitors regimens. Shorter durations of tamoxifen and aromatase inhibitor regimens are depicted using more translucent lines. Aromatase inhibitors with ovarian function suppression therapy was applied only for 5 years for the premenopausal breast cancer patients. The aromatase inhibitors for 10 years line in the subfigure for ‘All ages’ uses only 5 years of endocrine therapy for the premenopausal women with breast cancer.

There was a noticeable separation between the cumulative risk curves for contralateral breast cancer for the three durations of endocrine therapy, both for tamoxifen and for aromatase inhibitors (Fig. 4). The cumulative risk curve for 10-year tamoxifen therapy was similar to the cumulative risk curve for the 5-year aromatase inhibitor therapy. However, the features of cumulative risk of death from breast cancer were different, where 5-year and 10-years of aromatase inhibitor therapy resulted in similar risk curves but they were lower than the risk curves for 10-year tamoxifen therapy (Fig. 5). Moreover, the separation between the cumulative risk curves for contralateral breast cancer was wider than the corresponding set of curves for the risk of breast cancer death.

## Discussion

This is the first study to use an established simulation model to quantify the lifetime risk of contralateral breast cancer among the US female population with primary non-metastatic ER+/HER2- cancer and to provide estimates within 56 sub-groups based on primary endocrine therapy type, duration, and age at the diagnosis of the first primary cancer. Compared to tamoxifen, the aromatase inhibitor regimens produce a larger reduction in the number of contralateral breast cancers for all durations of endocrine therapy and ages. Across all regimes and durations, as expected, the difference in the number of avoided contralateral breast cancers was highest for women younger than 45 years at the diagnosis of their first primary cancer and decreased gradually with the increasing age at diagnosis. The cumulative risk of contralateral breast cancer over time after the primary diagnosis varied by the duration of endocrine therapy for the primary cancer. Overall, these model data provide a synthesis and extension of clinical trial data by presenting data about risks of contralateral breast cancer for different combinations of regimens, ages at primary diagnosis, and durations of use in the context of competing breast and other-cause mortality risks. Typically, the findings about the impact extended therapy and incomplete regimens on contralateral breast cancer are reported in separate clinical trials or meta-analyses. It is challenging to integrate the complexity across those studies to refine the estimates for specific age groups combined with endocrine therapy type and duration. CISNET’s breast cancer microsimulation model have already integrated and validated the influence of birth cohort, breast density trajectories, screening schedule on the 1st primary breast cancer, and subsequent dissemination of adjuvant therapy. Therefore, we could add this new layer of complexity for contralateral breast cancer for a more comprehensive simulation of life histories of women with breast cancer.

The results of this modeling study are consistent with and extend the results of clinical trials^3,6,15,16,24^. Our results also align with American Society of Clinical Oncology guidelines^5^ on adjuvant endocrine therapy for women with ER+ breast cancers and underscore the importance of preventing contralateral breast cancer as a key benefit of completing a full course of recommended endocrine therapy.

The risk of contralateral breast cancer is not the only, or the major, factor for selecting the endocrine therapy durations. Women and their clinicians may be interested in discussing avoidance of contralateral breast cancer in the context of overall survival and potential treatment side effects. For instance, while side effects of endocrine therapies will be largely abated with premature treatment termination, 2.5-year regimens increase the risk of breast cancer death and contralateral breast cancer across all age groups. Even for women whose ER+/HER2- cancer was diagnosed at age 75+ years, a 2.5-year regimen underachieves the potential reduction in contralateral breast cancer (vs. 5-year regimens). Current treatment decision tools (e.g., RSClin) provide data on the risk of primary cancer recurrence and breast cancer death, but do not also consider the cumulative risk of other-cause death and contralateral breast cancer^25^. In addition to informing clinical guidelines, well-established, validated simulation models can provide a powerful engine for development of clinical decision tools that synthesize multiple outcomes among groups of women with hundreds of unique combinations of individual, tumor, and treatment possibilities. The model used in this study has already been used as a platform to develop a screening^26^ and a treatment decision aid^22,27^.

Individual decisions about the type and duration of endocrine therapy depend on which outcomes a patient prioritizes. Most available data focus on the risk of breast cancer death or metastasis, as these are the primary concerns for most women. However, for some individuals, quality of life, the risk of contralateral breast cancer, and the possibility of facing another diagnosis may also be important. We hope clinicians and patients can use this study’s findings, alongside existing decision-making tools such as NHS Predict and RSClin, to make the most informed and personalized treatment choices.

Our results were consistent with SEER population trends and extend past studies to understand the lifetime tradeoffs of duration and type of endocrine therapy on contralateral breast cancer, overall life years, and the impact of therapy on at different ages. However, there are several limitations that should be considered in evaluating our results. First, the models provide data for average populations based on age at primary diagnosis and duration of treatment. While these data could be useful for informing or supporting clinical guidelines and discussions, results for individual women will vary, and further work would be needed to develop individual clinical decision tools similar to those developed in our past research^22,26,27^. We also did not specifically model results for women from racially or ethnically minoritized populations. Additionally, the trade-offs for stopping therapy based on immediate adverse effects vs. lowering the chance of developing contralateral breast cancer are highly variable and depend on many individual personal and contextual factors^28–31^. An analysis of patient-reported outcomes demonstrated that the reduction in health-related quality of life increases the risk of discontinuation of endocrine therapy, and recommended that women be assisted with clinical interventions to mitigate adverse events^12^. Another limitation is that we did not consider scenarios that included switching between tamoxifen and aromatase inhibitors for treating the primary breast cancer. Switching could reduce or increase the number of prevented contralateral breast cancers depending on the sequence and timing of the changes^32^. Explicit modeling of surveillance screening modalities and application of surveillance strategies tailored by clinical risk tools could theoretically improve the detection of contralateral breast cancers. We did do not include these variables in our model since there is insufficient data at present to guide model input parameters^33,34^. This will be an important area for future model extensions as new data emerge^35^. Finally, we did not consider bilateral mastectomy among high-risk women as a general population strategy to reduce contralateral breast cancers^36–38^. Recent data indicate that the occurrence of contralateral breast cancer may substantially increase breast cancer mortality^39,40^, but the same study found that bilateral mastectomy does not reduce breast cancer mortality. It is plausible that endocrine therapy, through its modulation of systemic as well as local risk, may attenuate not only the risk of contralateral breast cancer but also its associated increased risk of primary breast cancer mortality^41,42^. Breast surgeons may offer bilateral mastectomy to women at particularly high risk of a second breast cancer, typically due to an inherited pathogenic variant or, in some cases, a strong family history. Since our study does not model subgroups based on genetic syndromes or other risk factors, our results should not be used to guide bilateral mastectomy decisions based on individual risk status. However, they can help inform discussions on general risk reduction with endocrine therapy, especially when compared to published data on contralateral breast cancer risk reduction with mastectomy. This may be useful for some patients facing this decision.

Overall, 10-year endocrine therapy regimens produce a reduction in contralateral breast cancer cases compared to 5-year regimens, especially among women who are younger vs. older at the diagnosis of their first breast cancer. Since, the reduction in breast cancer deaths due to extending endocrine therapy to 10 years is minimal, the decrease in contralateral breast cancers may provide a more compelling support for extended endocrine therapy for younger women with ER+/HER2- cancers. This analysis illustrates the use of simulation modeling to synthesize and extend clinical trial results to project multiple outcomes among groups of women with unique combinations of characteristics. These data could be used in the future to develop and test clinical decision tools to support conversations about contralateral breast cancer and other outcomes of interest to women with breast cancer.

## Methods

### Model overview

We used CISNET Model-GE (developed at Georgetown University and Albert Einstein College of Medicine) for this study. As the site of the Coordinating Center for the CISNET Breast Working Group, the University of Wisconsin Institutional Review Board determined that this study was *Not Human Subjects Research*. Model-GE has been previously used to inform US Preventive Services Task Force^19,23,43^, American Cancer Society^44^, CDC screening guidelines^45^, and estimating the impact of breast cancer screening and treatment^46^. The model has been described in detail elsewhere^47,48^. Briefly, Model-GE stochastically simulates life histories of multiple US population birth cohorts (Fig. 1 in Schechter et. al.^47^). Women may either develop breast cancer or not in their lifetime. Breast cancers have a molecular subtype-specific sojourn time and can be diagnosed via screening or clinical detection^49,50^. The stage of cancer is conditional on the age at diagnosis, molecular subtype and mode of detection^49,50^. The hazard of breast cancer death is reduced based on age-, stage- and subtype-specific treatment effects. Women may die of breast cancer or other causes based on age and birth cohort.

### Population

We simulated the US female population of multiple birth-year cohorts born between 1890–2010. Simulated women are followed from birth to death (or age 120) and the events women experience (e.g., primary breast cancer, contralateral breast cancer, cause of death) are collated starting in 1975. Based on observed population survival data with a tail of a small number of women who live beyond age 100, we use age 120 is a strict upper bound in our simulation input. Hence, the survival function for breast cancer, contralateral breast cancer, or non-breast cancer causes reaches zero at age 120.

### Model input parameters

Model GE input parameters have been summarized previously^51^. New model inputs are summarized in Table 1 and Supplementary Tables 1 and 2.

#### Contralateral breast cancer incidence

We used data from the Surveillance, Epidemiology, and End Results (SEER registry 8)^52^ and the contralateral breast cancer incidence hazard ratios from the Netherlands Cancer Registry^9^ to create a contralateral breast cancer incidence function for each type of primary adjuvant regimen, including a no adjuvant therapy category, where each primary regimen was conditional on age, stage, and ER/HER2 subtype at primary diagnosis^46,53^. We used the data from the SEER registry 8 (1975–2020) (Supplementary Method 1 and Supplementary Figs. 1 and 2) to derive the metachronous contralateral breast cancer incidence functions for all women with primary breast cancer (Fig. 2, Supplementary Method 2 and Supplementary Fig. 3)^52^. Women with bilateral mastectomy after the first primary breast cancer were excluded in calculation of the incidence rate of contralateral breast cancer. The Kaplan-Meier (KM) survival functions for contralateral breast cancer incidence cover shorter duration of years for latter years of 1st primary diagnosis (Fig. 2). The first observation is that the lifetime risk of contralateral breast cancer is high, nearly 18%. Secondly, the contralateral breast cancer incidence survival functions for different years of 1st primary diagnosis do not collapse into a single curve. For comparatively recent years of 1st primary diagnosis (1995 onwards), the risk of contralateral breast cancer has decreased in the first 10 years. But the risk of contralateral breast cancer is increasing faster near the 20-year mark than it used to for earlier years of 1st primary diagnosis (before 1995). The high lifetime risk of contralateral breast cancer coupled with the changing trend for the recent years of 1st primary diagnosis emphasize the need for our work.

Since the data in SEER represents the overall rates in the presence of all types of adjuvant therapies for the primary breast cancer in the survivor population, we computationally extracted the contralateral breast cancer incidence functions for the no adjuvant therapy group using hazard ratios from the Netherlands Cancer Registry and the fraction of the population with breast cancer in each adjuvant therapy group. We also adjusted the survival functions using the relative risks for contralateral breast cancer by stages of first primary breast cancer using data from SEER, using the proportional hazard model.

The contralateral breast cancer incidence hazard ratios for specific regimens (i.e., tamoxifen or aromatase inhibitors with or without ovarian suppression and with or without chemotherapy) and durations (none, 2.5, 5 and 10 years) of endocrine therapy for ER+/HER2- cancers were obtained from clinical trials and Netherlands population registry data^3,6,15,18,24,32,54^. The relatively lower risk reduction due to chemotherapy compared to endocrine therapy on contralateral breast cancer rates was also included in the model. We validated this approach by comparing observed SEER data to our model projected contralateral breast cancer incidence rates based on the incidence functions derived from the deconvolution of SEER data and use of the Netherlands data.

#### Treatment

The competing risk of primary breast cancer death was based on US treatment patterns for 14 combinations of different endocrine regimens and chemotherapy by year, age, ER/HER2, stage at diagnosis, and the efficacy of those therapies reported in a meta-analyses of clinical trials^51^ conditional on age, ER/HER2 subtype, and stage, as used in previous CISNET BWG models^19,46,53,55^. For simplification, we assumed in each simulation scenario that all women received a single type of primary endocrine therapy regimen within the primary treatment duration period, with exceptions due to the standard of care. Premenopausal patients with Stage 0 or Ductal Carcinoma in Situ (DCIS) cancer were given tamoxifen therapy with a maximum duration of 5 years. Premenopausal women receiving ovarian function suppression (with tamoxifen or aromatase inhibitors) were given the treatment for a maximum of 5 years. Endocrine therapy increases the risk of cardiovascular events, musculoskeletal symptoms, osteoporosis and fractures, but the increase in other-cause mortality rate due to these adverse events is low and neglected in this study^6,10,56^.

For treatment of contralateral breast cancer, we assumed the same treatment dissemination and efficacy as for the 1st primary breast cancer. Based on clinical opinion, we assumed the duration of the endocrine treatment for ER+ contralateral breast cancer was 5-years. Additionally, for ER+ contralateral breast cancers occurring within <5-years of the primary cancer, we assumed that if the original endocrine therapy was tamoxifen, contralateral breast cancer therapy would be switched to an aromatase inhibitor regimen (with ovarian function suppression for premenopausal women), and if the primary breast cancer treatment was with aromatase inhibitors, we assumed that the contralateral breast cancer treatment was switched to tamoxifen in 50% of cases and the other 50% received aromatase inhibitors.

#### Non-cancer mortality

Age and birth cohort-specific non-cancer mortality was based on US life tables^57^.

#### Outcomes

The primary outcomes included the number of life time contralateral breast cancers, breast cancer deaths and other-cause deaths by age at the diagnosis of the first breast cancer. Secondary outcomes included the cumulative probability (or risk) of contralateral breast cancer and breast cancer death with time since the diagnosis of the first primary. We also estimated the QALYS due to different durations of aromatase inhibitors therapy to assess the impact of adverse events on the quality-of-life of breast cancer survivors (last section of “Methods”).

### Analysis

We analyzed the lifetime outcomes under 12 scenarios based on combinations of three endocrine regimens: aromatase inhibitor (with mandatory ovarian function suppression for pre-menopausal women), tamoxifen, and tamoxifen with ovarian function suppression for pre-menopausal women and four endocrine therapy durations: none, 2.5, 5 and 10 years. We also separated the analysis into six age groups (all, <45, 45–49, 50–64, 65–74, and 75+ years). We simulated 400 million women in each scenario to minimize differences due to stochastic variation. Outcomes were summarized as number of events per 100 women diagnosed with primary ER+/HER2- breast cancer and number of events for a given therapy duration vs. no endocrine therapy. The numbers were reported for per 100 women for the ease of communication in clinical settings. Simulation results were tallied for women whose first breast cancer was diagnosed between the age of 30–79 years between 1975 and 2050. All relevant life history events (i.e., first breast cancer, contralateral breast cancer, deaths) were only included in the tally if they occurred between 1975 and 2050. Finally, we estimated the cumulative probabilities of developing a contralateral breast cancer and death due to the primary breast cancer, for different treatment regimens as a function of time since the diagnosis of the first primary.

### Validation

The overall modeled contralateral breast cancer incidence rate was calculated for women with first primary ER+ breast cancer. Model GE includes different incidence rates for each of the 14 adjuvant endocrine and chemotherapy (and no therapy) regimens, but SEER does not report these data so, we cannot do a direct regimen-specific comparison. Therefore, we compared the overall contralateral breast cancer incidence to the observed overall SEER-12 rates from 1996–2020. We selected this period to allow sufficient time to develop a contralateral breast cancer after SEER began collecting ER data in 1990. We also explored the model vs. SEER rates considering ER+/HER2-, but HER2 data were added to SEER only from 2010, so there has only been a limited time period for development and observation of contralateral breast cancer by HER2 status.

### Obtaining metachronous contralateral breast cancer data from SEER

To simulate the incidence of contralateral breast cancer in the life history of a breast cancer survivor, we need a set of contralateral breast cancer incidence functions as input, $S\left({t;}\left\{c\right\}\right)$, where $t$ is the *latency* or the time interval between the 1st primary event and the contralateral breast cancer diagnosis, and $\left\{c\right\}$ is a set of variables that specifies the category of breast cancer survivors: (a) year of 1st primary diagnosis, (2) age at the 1st primary diagnosis, and (3) the treatment for the 1st primary. The function $S\left({t;}\left\{c\right\}\right)$ is used to sample the latency value while stochastically simulating the life history of a person.

To construct the contralateral breast cancer incidence functions, $S\left({t;}\left\{c\right\}\right)$, we used the SEER registry 8 database that contains breast cancer surveillance data from 1975 to 2020 for the US population. We collected the metachronous contralateral breast cancer incidence data categorized by the year of first primary diagnosis, i.e., for each year from 1975 to 2020. We further categorized the data in the following age groups: 40–44, 45–49, 50–64, 65–74, and 75+ years. We chose these age groups because the 1st primary input data for the CISNET models also uses these groups. Supplementary Fig. 1 shows the incidence rate obtained from SEER8, the y-axis is the year of first primary diagnosis, and the x-axis is the latency. For more recent years of 1st primary diagnosis, we have shorter latency period for which SEER has been able to surveil breast cancer survivors for contralateral breast cancer cases. Therefore, the lower half of Supplementary Fig. 1, below the right diagonal, is zero.

Since we have collected the incidence rate with a short time interval, 1-year window in latency and 1-year window in the year of diagnosis, the incidence rate from SEER has additional stochasticity due to small number of samples. To correct for this stochasticity, we smoothed the incidence rate data in Supplementary Fig. 1 using a moving average method. For each cell (latency, year of 1st primary diagnosis)-coordinate in Supplementary Fig. 1, we selected a window of 5-by-5 years centered around the cell and used the average incidence rate for all cells within that window as the value for the central cell. Of course, the cells in the moving average window that were below the right diagonal were excluded from the moving average calculation. The smoothed contralateral breast cancer incidence rate field is shown in Supplementary Fig. 2. This smoothed incidence rate was used to calculate the KM survival functions for contralateral breast cancer incidence shown in Fig. 2 of the main text.

### Extending the latency domain for the contralateral breast cancer incidence functions from SEER

We used the smoothed incidence rate data, as shown in Supplementary Fig. 2, to determine the contralateral breast cancer incidence functions using the KM estimator, which is shown in Fig. 2. We need the contralateral breast cancer incidence functions for all years of 1st primary diagnosis to cover a sufficiently long latency range, 50 years, so that we can cover the residual lifetime of breast cancer survivors. We extended the contralateral breast cancer incidence functions in Fig. 2 by fitting against the improper Gompertz survival function, which is shown below.

1 $$S\left(t\right)=c+\left(1-c\right)\exp (-a({e}^{{bt}}-1))$$

We chose the Gompertz survival function because it has been found to be the best function for modeling tumor growth^58,59^. We used improper Gompertz function with a non-zero cure fraction, i.e., $c\, > \,0$, because the contralateral breast cancer incidence functions do not reach zero with increasing latency, as all breast cancer patients do not experience a contralateral breast cancer event in their lifetime. We obtained the parameters *a*, *b*, and *c* by fitting the CBC incidence functions in Supplementary Fig. 3 to the above equation. Then we obtained $S(t)$ for latency $t\in [\mathrm{0,50}]$ years, to get contralateral breast cancer incidence functions for each year of 1st primary diagnosis from 1975 to 2010 and each of them covering 50 years of latency. We used the contralateral breast cancer incidence function from 1975 for years earlier than 1975, and we used the contralateral breast cancer incidence function from 2010 for years after 2010.

### Transforming the contralateral breast cancer incidence functions from the no adjuvant therapy to the any adjuvant therapy population group

The extended contralateral breast cancer incidence functions are of the form, *S(t; age at 1st* *BC, year of 1st* *BC diagnosis)*. But we also need the contralateral breast cancer incidence functions to depend on the type of 1st primary therapy. We used the adjuvant therapy-specific contralateral breast cancer incidence hazard ratios from the NCR, which uses *no adjuvant therapy* population as the reference (control) group^9^. The breast cancer patient population surveilled in SEER is a mixture of subpopulations that have received different types of adjuvant therapies (or none), we label it as the *any adjuvant therapy* population. Moreover, the proportion of these subpopulations have evolved from 1975–2020. So, we cannot directly apply the contralateral breast cancer incidence hazard ratios from NCR to the extended contralateral breast cancer incidence functions that we derived from SEER. We need to transform the contralateral breast cancer incidence hazard ratios from the *no adjuvant therapy* reference group to the *any adjuvant therapy* reference group and we performed this transformation using the following method.

Let ${q}_{{mixed}}(T)$ be the contralateral breast cancer incidence rate for the year $T$ for all 1st primary breast cancer survivors, i.e., the *any adjuvant therapy* group. Let ${q}_{\theta }(T)$ be the CBC incidence rate for the year $T$ for all 1st primary breast cancer survivors who received the therapy $\theta$. Then the following relationship holds

2 $${q}_{{mixed}}(T)=\sum _{\theta \in \Theta }{q}_{\theta }{\left(T\right)\rho }_{\theta }(T)$$

Where ${\rho }_{\theta }(T)$ is the fraction of 1st primary breast cancer survivor who received the treatment type $\theta$ and $\Theta$ is the set of all type of treatments, including no adjuvant therapy. If the CBC incidence hazard ratio for treatment type $\theta$ with respect to the any adjuvant therapy reference group is ${\lambda }_{\theta }$, then

3 $${q}_{\theta }(T)={\lambda }_{\theta }(T){q}_{{mixed}}(T)$$

Using the above two equations we obtain the self-consistency criterion for contralateral breast cancer incidence hazard ratios with the any adjuvant therapy reference group as

4 $$\sum _{\theta \in \Theta }{\lambda }_{\theta }{\left(T\right)\rho }_{\theta }\left(T\right)=1$$

Let ${\lambda }_{\theta }^{({no}-{adj})}$ be the contralateral breast cancer incidence hazard ratio with respect to the no adjuvant therapy group, which we obtained from NCR, and let $K(T)=\sum _{\theta \in \Theta }{\lambda }_{\theta }^{\left({no}-{adj}\right)}(T)$. Then, ${\lambda }_{\theta }\left(T\right)={\lambda }_{\theta }^{\left({no}-{adj}\right)}/K(T)$, is the contralateral breast cancer incidence hazard ratio with respect to the mixed therapy group that satisfies Eq. (4). We applied the contralateral breast cancer incidence hazard ratios, ${\lambda }_{\theta }(T)$, on the contralateral breast cancer incidence functions from SEER (Supplementary Fig. 3), to get CBC incidence functions that depend on the type of treatment for the 1st primary.

We also made the contralateral breast cancer incidence survival functions sensitive to the stage of the first primary breast cancer. We obtained from SEER the overall contralateral breast cancer incidence rate between years 2010–2021 among women with different stages of first primary breast cancer. We used the data for contralateral breast cancer incidence in Stage I, II, and III patients only, because the number of contralateral breast cancer cases in Stage 0 and Stage IV survivors were small. We determined the relative risk of contralateral breast cancer incidence for Stage I, II, and III with respect to the overall contralateral breast cancer incidence rate and for Stage 0 and IV we assumed a relative risk of 1 (vs. the overall, i.e., any stage, contralateral breast cancer incidence rate). We applied this set of relative risk values to the treatment-dependent contralateral breast cancer incidence survival functions using the proportional hazard model.

### Quality-adjusted life years (QALYS) gained due to different durations of aromatase inhibitors therapy

We estimated the impact of adverse events due to aromatase inhibitors on the quality-of-life of breast cancer survivors. We collected the data for the prevalence rates for the 4 adverse events associated with endocrine therapy, musculoskeletal symptoms, osteoporosis, fracture, and endometrial cancer, with the 95% confidence interval for each of the prevalence rates (Supplementary Table 3). We chose three levels of disutility values for each of these adverse events for a parametric estimation of QALYS due to different durations of aromatase inhibitors therapy. The quality adjustment due to adverse events were applied both during the treatment of the first primary breast cancer and the contralateral breast cancer, if any. We also included the age-dependent and breast cancer stage-dependent disutility in the calculation of QALYs.

The QALYs for 5 years of aromatase inhibitors therapy with the mean prevalence rates for adverse events and mean level of disutility was used as a reference value. The increase or decrease in QALYs for other durations of aromatase inhibitors therapy with different combinations of prevalence rates and disutility is shown in Supplementary Fig. 5. The sensitivity analysis shows that 10-year aromatase inhibitors regimen can provide a gain in QALYs (vs 5-year aromatase inhibitors therapy), only if the prevalence rates and disutility of adverse events is low.

The prevalence rates data for adverse events of endocrine therapy varies across multiple studies, and it is also challenging to determine the right choice of disutility values. Therefore, the main purpose of Supplementary Fig. 5 is to qualitatively show that a net gain in QALYs for 10-year of aromatase inhibitors can be difficult without treatments to reduce the symptoms from adverse events. Including other adverse events of aromatase inhibitors therapy in the QALYs calculation can change the numbers but it would not change this main conclusion.

## Supplementary information

Supplementary Figures and Tables

## Data Availability

Detailed information about the CISNET model used in this analysis is available online at https://cisnet.cancer.gov/resources/documentation.html. Data used in this analysis come from published manuscripts and publicly available reports. Some data are not available from the authors but can be obtained from the original sources, including previously published estimates of screening and treatment dissemination that relied on data from the Breast Cancer Surveillance Consortium and the National Comprehensive Cancer Center Network, respectively. Researchers may enter into data use agreements with these entities (and their data sources, when required by state law) to obtain these data.

## Code availability

The underlying code used for this study is not publicly available but maybe made available to qualified researchers on reasonable request from the corresponding author.
