# Supplementary material for: Impact of endocrine therapy regimens for early-stage ER+/HER2- breast cancer on contralateral breast cancer risk
Source: NPJ Breast Cancer. 2025 Mar 26;11:30. doi: 10.1038/s41523-025-00746-7 (PMC11947086; doi:10.1038/s41523-025-00746-7)
Supplement: Supplementary file 1 — Supplementary Figures and Tables [file 41523_2025_746_MOESM1_ESM.pdf]

Supplementary Figures and Tables: Impact of Endocrine Therapy Regimens for Early-Stage ER+/HER2- Breast Cancer on Contralateral Breast Cancer Risk

Swarnavo Sarkar[1], Clyde Schechter[2], Allison W. Kurian[3], Jennifer L. Caswell-Jin[3], Jinani Jayasekera[4] and Jeanne S. Mandelblatt[1]

[1]Lombardi Comprehensive Cancer Center, Washington, District of Columbia, USA
[2]Albert Einstein College of Medicine, New York City, New York, USA
[3]Stanford University School of Medicine, Stanford, California, USA
[4]National Institutes of Health, Rockville, Maryland, USA

Supplementary Figures:

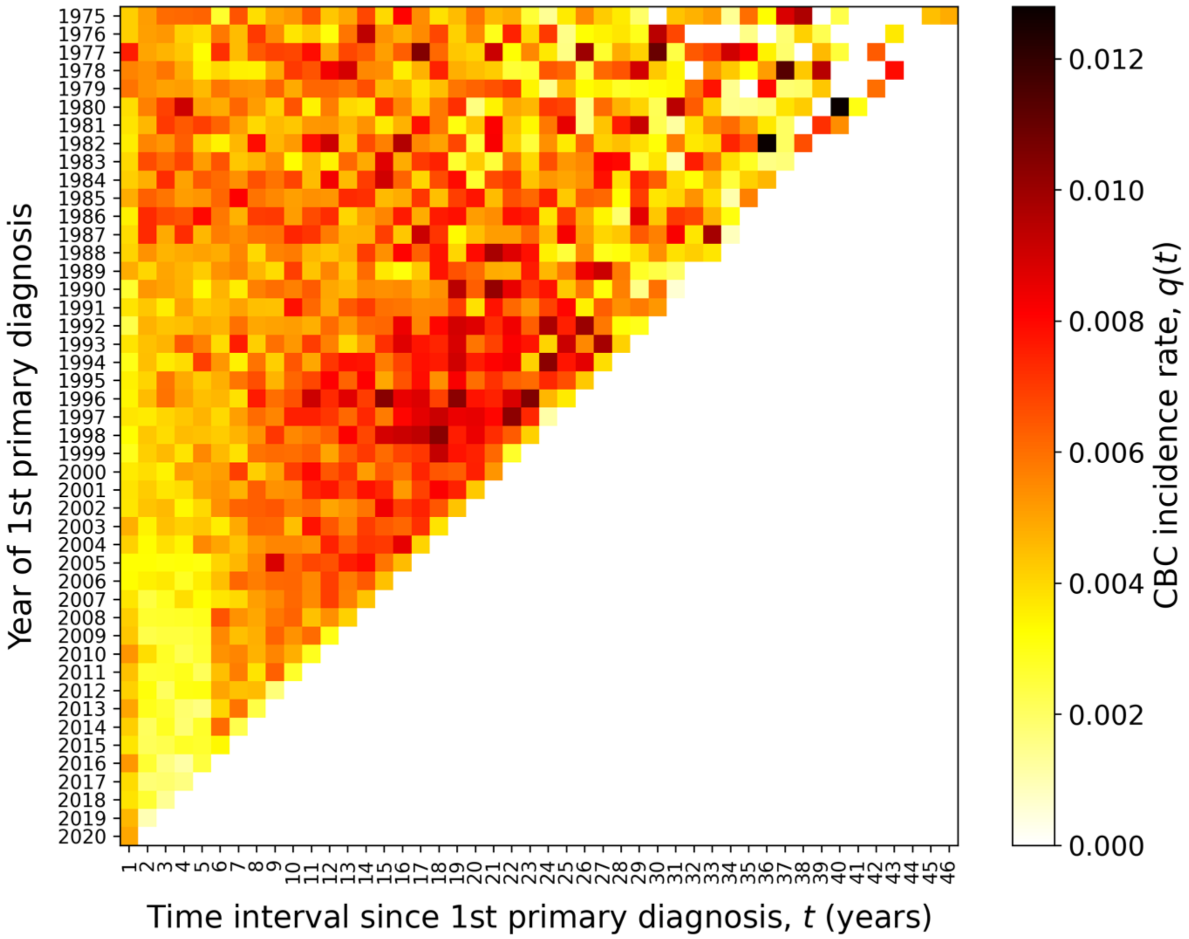

Supplementary Figure 1: Contralateral breast cancer incidence rate for the year of 1st primary diagnosis and the time interval after the 1st primary diagnosis (latency). This figure shows the incidence rate for all ages. But for our model inputs we collected the data further categorized by the age groups of 40-44, 45-49, 50-64, 65-74, and 75+.

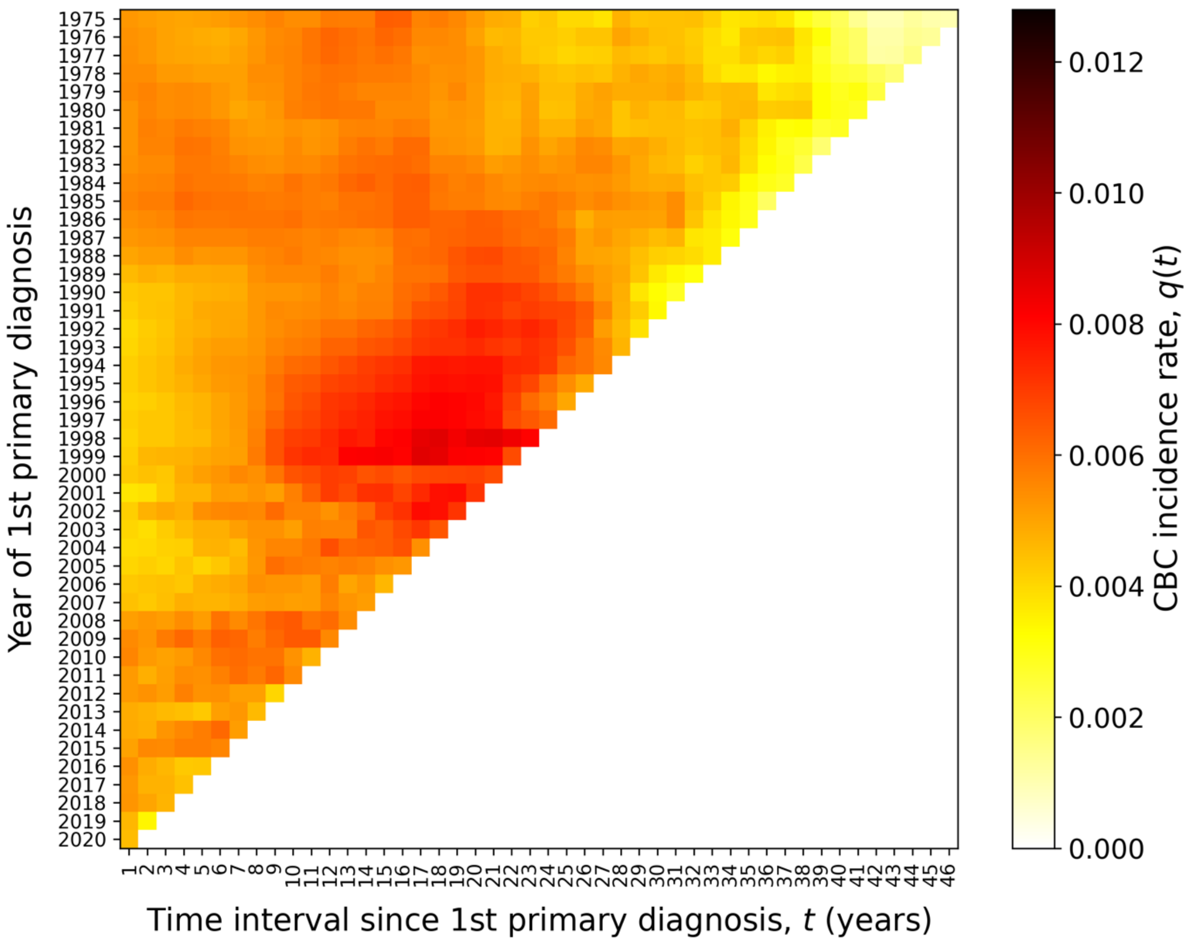

Supplementary Figure 2: Smoothed incidence rate for contralateral breast cancers. The average incidence rate in a neighborhood of 5-by-5 cells centered around each cell was assigned as the value of the central cell. This figure shows the incidence rate for all ages. We used the similar moving average method to determine the smoothed CBC incidence rate for the age groups of 40-44, 45-49, 50-64, 65-74, and 75+.

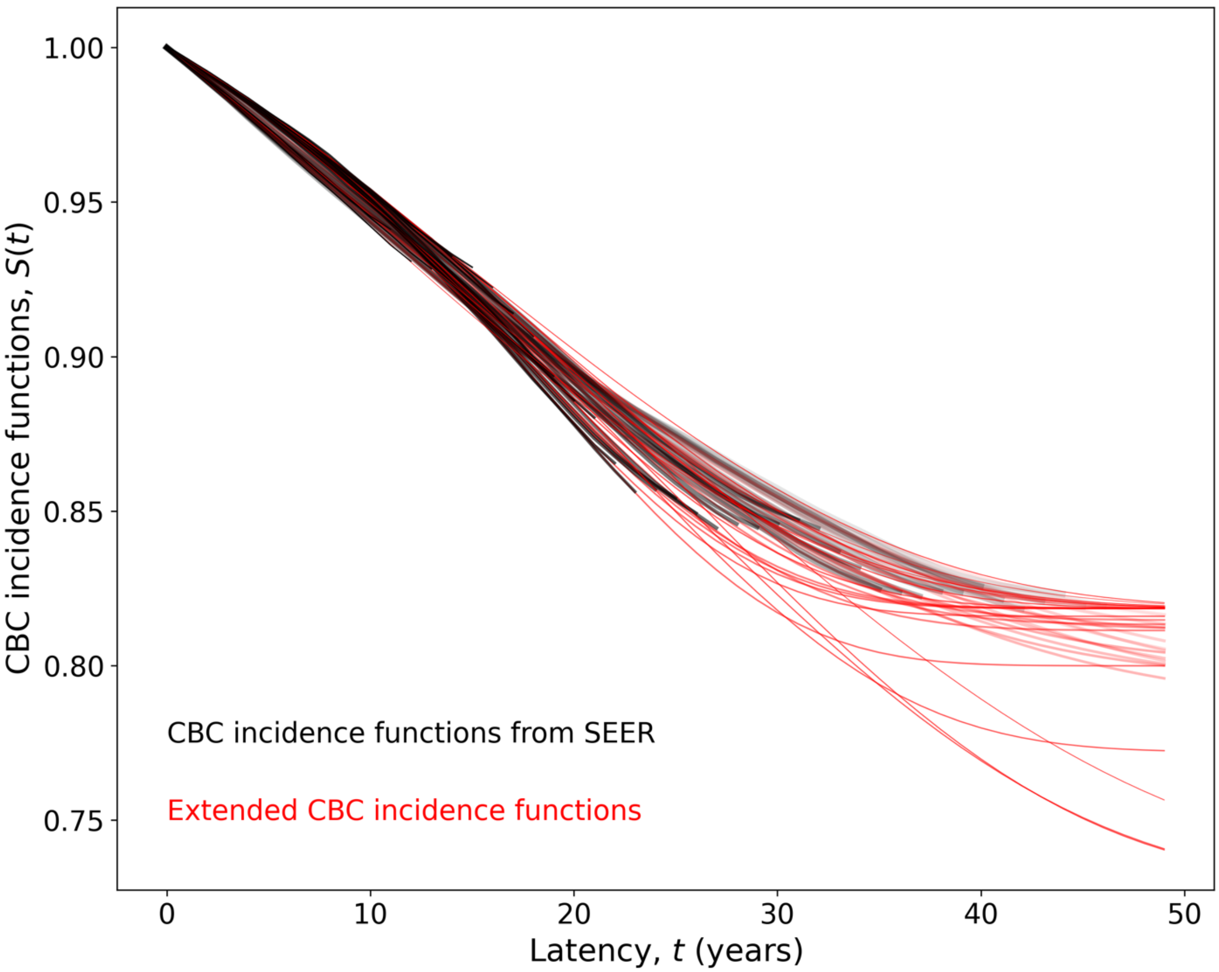

Supplementary Figure 3: Extended CBC incidence survival functions using the improper Gompertz survival function model. The black lines are the same CBC incidence survival functions from Figure 2 in the manuscript. The red lines are the extended CBC incidence survival functions that covers the latency of $t \in [0,50]$ years. We used this same approach to extend the CBC incidence survival functions for the 5 different age groups of 40-44, 45-49, 50-64, 65-74, and 75+ years.

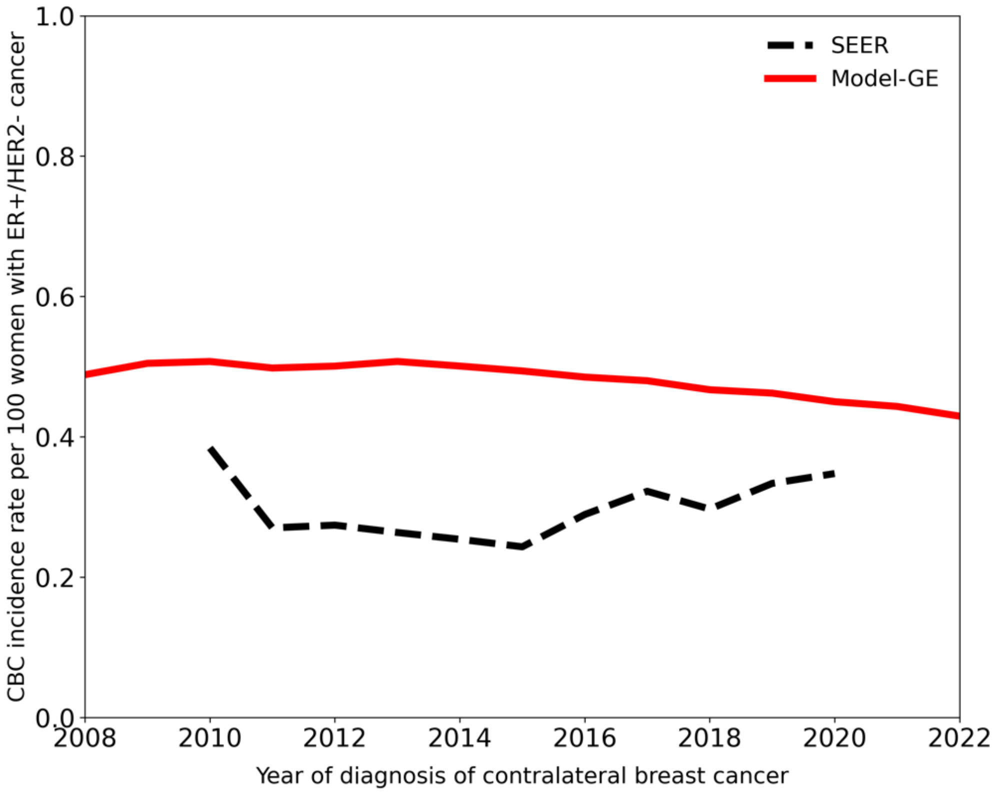

Supplementary Figure 4: Comparison of contralateral breast cancer incidence rate from Model-GE with the SEER data for ER+/HER2- survivors. SEER began surveilling HER2 subtype only from 2010. Therefore, the ER+/HER2- population surveilled for contralateral breast cancer is low and has been surveilled for only 10 years of latency, which is probably insufficient to estimate the true contralateral breast cancer incidence rate in ER+/HER2- survivors.

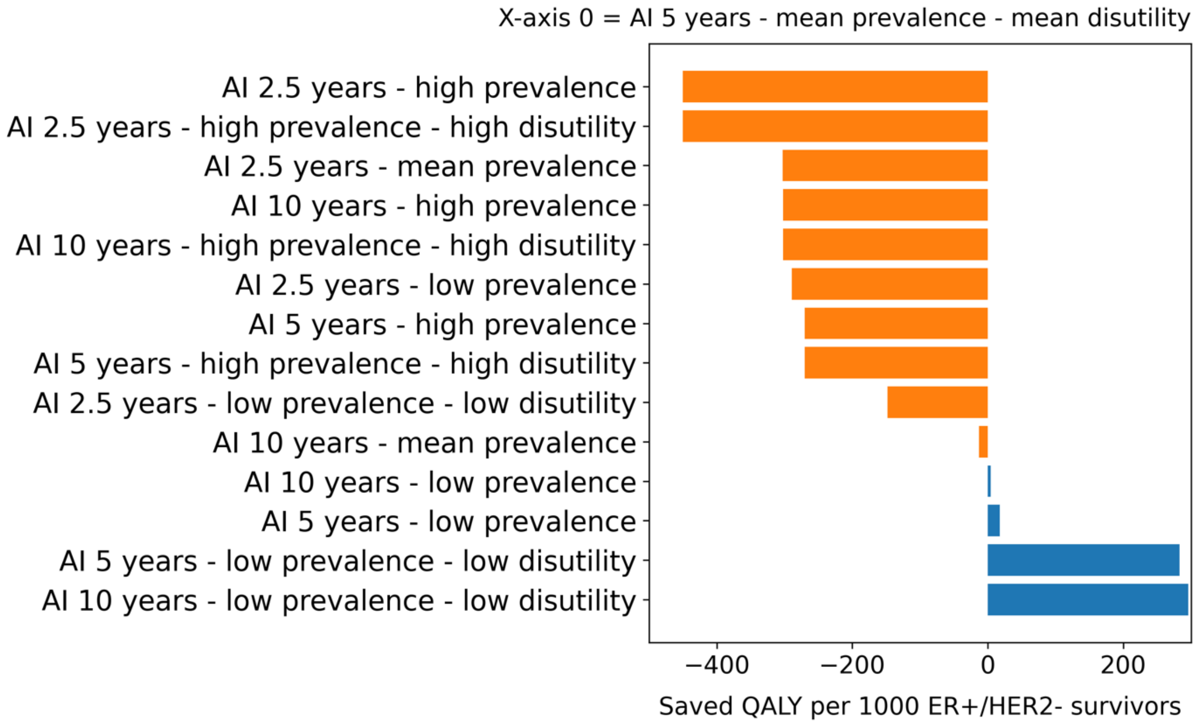

Supplementary Figure 5: Sensitivity analyses of quality-adjusted life years. Quality-adjusted life years for different aromatase inhibitors therapy regimens were computed using the confidence interval values for the prevalence rates and three levels of disutility weights for the four adverse events (included in Supplementary Table 3). The x-axis zero or the baseline value is the absolute number of quality-adjusted life years for all ages from 5 years of aromatase inhibitors therapy using the mean prevalence and mean disutility weights. The y-axis labels show the combination of prevalence rates and disutility values used to compute the QALYs for aromatase inhibitors therapy regimens. One-way sensitivity analysis uses either higher (lower) values of prevalence rates or disutility weights, like AI 5 years – low prevalence. Multi-way sensitivity analysis uses higher (lower) values for both the prevalence rates and disutility weights, like AI 10 years -high prevalence – high disutility.

Supplementary Tables:

Supplementary Table 1: Overall survival and CBC incidence hazard ratios for 3 different durations of tamoxifen therapy.

Data type			Data sources
**For specific ET and adherence**			
Overall survival HRs	Tamoxifen, 2.5 years	0.9	5-year HRs from the EBCTCG data[1].
Age < 50 years	Tamoxifen, 5 years	0.68	2.5-year HRs from the non-adherence HRs
	Tamoxifen, 10 years	0.59	in the Kaiser Permanente of Northern
	Tamoxifen, 2.5 years	0.88	California data[2].
Age >= 50 years	Tamoxifen, 5 years	0.67	10-year HRs from the ATLAS trial data[3].
	Tamoxifen, 10 years	0.58	
Contralateral breast cancer			
incidence HRs	Tamoxifen, 2.5 years	0.86	
Age < 50 years	Tamoxifen, 5 years	0.67	
	Tamoxifen, 10 years	0.5	
	Tamoxifen, 2.5 years	0.84	
Age >= 50 years	Tamoxifen, 5 years	0.46	
	Tamoxifen, 10 years	0.345	

Supplementary Table 2: Overall survival and CBC incidence hazard ratios for 3 different durations of tamoxifen + OFS therapy.

Data type			Data sources
**For specific ET and adherence**			
Overall survival HRs	Tamoxifen + OFS, 2.5 years	0.58	5-year HRs from SOFT-TEXT trials[4,5].
Age < 50 years	Tamoxifen + OFS, 5 years	0.46	2.5-year HRs from the non-adherence HRs
	Tamoxifen + OFS, 10 years	-	in the Kaiser Permanente of Northern
Contralateral breast			
cancer incidence HRs	Tamoxifen + OFS, 2.5 years	0.74	California data[2].
Age < 50 years	Tamoxifen + OFS, 5 years	0.58	
	Tamoxifen + OFS, 10 years	-	

Supplementary Table 3: The prevalence rates and disutility weights for the adverse events were used to calculate the quality-adjusted life years for different durations of aromatase inhibitors therapy.

Input parameter	Data			
**Prevalence rates (95% CI) for selected adverse events of aromatase inhibitors therapy for primary breast cancer**				
	Treatment duration			Data sources: SOFT-TEXT trials[4,5] ATLAS trial[3] IDEAL trial[6] ATAC trial[7]
Adverse Event	2.5 years	5 years	10 years	
Musculoskeletal symptoms	44.5% (43.5% - 45.5%)	89.9% (87.9% - 91.9%)	90% (88% - 92%)	
Osteoporosis	21.1% (20.1% - 22.1%)	42.2% (40.2% - 44.2%)	54.2% (52.2% - 56.2%)	
Fracture	3.85% (3.35% - 4.35%)	7.7% (6.7% - 8.7%)	14% (12.5% - 15.5%)	
Endometrial cancer	0.32% (0.27%. - 0.37%)	0.32% (0.27%. - 0.37%)	0.59% (0.55% - 0.63%)	
**Disutility weights for the adverse events**				
Adverse event	Disutility weights			
	Mean	Low	High	
Musculoskeletal symptoms	0.1	0.05	0.15	
Osteoporosis	0.1	0.05	0.15	
Fracture	0.05	0.025	0.075	
Endometrial cancer	0.2	0.1	0.3	
**Age and breast cancer stage dependent disutility weights**				
Age-specific disutility	Values from the reference.	Hanmer *et. al.*[8]		
Cancer-specific disutility	Values from references.	De Haes et. al.[9] and Stout et. al.[10]		

Supplementary References:

1. EBCTCG. Tamoxifen for early breast cancer:an overview of the randomised trials. *Lancet* vol. 315 1451–1467 (1998).
2. Hershman, D. L. *et al.* Early discontinuation and non-adherence to adjuvant hormonal therapy are associated with increased mortality in women with breast cancer. *Breast Cancer Res. Treat.* **126**, 529–537 (2011).
3. Davies, C. *et al.* Long-term effects of continuing adjuvant tamoxifen to 10 years versus stopping at 5 years after diagnosis of oestrogen receptor-positive breast cancer: ATLAS, a randomised trial. *The Lancet* **381**, 805–816 (2013).
4. Francis, P. A. *et al.* Tailoring Adjuvant Endocrine Therapy for Premenopausal Breast Cancer. *N. Engl. J. Med.* **379**, 122–137 (2018).
5. Pagani, O. *et al.* Adjuvant Exemestane With Ovarian Suppression in Premenopausal Breast Cancer: Long-Term Follow-Up of the Combined TEXT and SOFT Trials. *J. Clin. Oncol.* **41**, 1376–1382 (2023).
6. Blok, E. J. *et al.* Optimal Duration of Extended Adjuvant Endocrine Therapy for Early Breast Cancer; Results of the IDEAL Trial (BOOG 2006-05). *JNCI J. Natl. Cancer Inst.* **110**, 40–48 (2018).
7. Anastrozole alone or in combination with tamoxifen versus tamoxifen alone for adjuvant treatment of postmenopausal women with early breast cancer: first results of the ATAC randomised trial. *The Lancet* **359**, 2131–2139 (2002).
8. Hanmer, J. & Kaplan, R. M. Update to the Report of Nationally Representative Values for the Noninstitutionalized US Adult Population for Five Health-Related Quality-of-Life Scores. *Value Health* **19**, 1059–1062 (2016).

9. De Haes, J. C. J. M. *et al.* The impact of a breast cancer screening programme on quality-adjusted life-years. *Int. J. Cancer* **49**, 538–544 (1991).

10. Stout, N. K. *et al.* Retrospective Cost-effectiveness Analysis of Screening Mammography. *JNCI J. Natl. Cancer Inst.* **98**, 774–782 (2006).
